# The Influence of Infective Dose on the Virulence of a Generalist Pathogen in Rainbow Trout (*Oncorhynchus mykiss*) and Zebra Fish (*Danio rerio*)

**DOI:** 10.1371/journal.pone.0139378

**Published:** 2015-09-30

**Authors:** Hanna Kinnula, Johanna Mappes, Janne K. Valkonen, Lotta-Riina Sundberg

**Affiliations:** Centre of Excellence in Biological Interactions, Department of Biological and Environmental Science, University of Jyvaskyla, Finland; University of Texas Medical Branch, UNITED STATES

## Abstract

Pathogen density and genetic diversity fluctuate in the outside-host environment during and between epidemics, affecting disease emergence and the severity and probability of infections. Although the importance of these factors for pathogen virulence and infection probability has been acknowledged, their interactive effects are not well understood. We studied how an infective dose in an environmentally transmitted opportunistic fish pathogen, *Flavobacterium columnare*, affects its virulence both in rainbow trout, which are frequently infected at fish farms, and in zebra fish, a host that is not naturally infected by *F*. *columnare*. We used previously isolated strains of confirmed high and low virulence in a single infection and in a co-infection. Infection success (measured as host morbidity) correlated positively with dose when the hosts were exposed to the high-virulence strain, but no response for the dose increase was found when the hosts were exposed to the low-virulence strain. Interestingly, the co-infection resulted in poorer infection success than the single infection with the high-virulence strain. The rainbow trout were more susceptible to the infection than the zebra fish but, in both species, the effects of the doses and the strains were qualitatively similar. We suggest that as an increase in dose can lead to increased host morbidity, both the interstrain interactions and differences in infectivity in different hosts may influence the severity and consequently the evolution of disease. Our results also confirm that the zebra fish is a good laboratory model to study *F*. *columnare* infection.

## Introduction

Virulence (the harm caused to the host by a pathogen) is influenced by several ecological and evolutionary processes that often involve a trade-off between host exploitation and pathogen reproduction [1, 2]. The key factors driving the virulence of a pathogen (e.g. host susceptibility, pathogen growth rate and host-specificity) coevolve in an arms race between the pathogen and its host [1]. Opportunistic pathogens are often host-generalists and can have the ability to survive and replicate outside the host, thus not being restricted by the transmission-virulence trade-off [3]. Despite the opportunists having a great impact on general health, their infection dynamics and infectivity in different host species are poorly characterized, and not covered by the traditional theories of virulence.

In nature, populations of hosts and their pathogens are diverse, and the hosts are often infected by several pathogen genotypes or species, which often leads to increased virulence [4, 5, 6, 7]. Interactions of co-infecting pathogens can have a significant role in virulence evolution, either due to strain competition or by facilitating infection with cooperative interactions [4, 8, 9, 10]. Co-infections may be especially important for generalist pathogens that have a wide host range and, thus, a higher likelihood of coming across potential hosts than host-specialists [3, 11]. However, pathogen virulence may face trade-offs as a result of the ecological and evolutionary costs of generalism [12], which could lead to higher pathogen doses needed for the initiation of an infection. As a consequence, the virulence of a generalist pathogen in different hosts may not always be easy to predict [11].

Infective dose (the number of cells needed to infect a host) varies greatly among pathogen species [13]. The infective dose is generally recognized to influence disease dynamics and severity [14, 15], as virulence typically increases with the dose [8, 14]. Although dose effects in multiply infected hosts can have important evolutionary consequences [16], the strain interactions in the context of dose effect are still poorly understood.

Previous studies on co-infections with eukaryotic parasites have demonstrated the virulence increase in the fish pathogen *Flavobacterium columnare* [17, 18]. However, it has remained unknown how the interstrain interactions of different *F*. *columnare* strains affect the virulence during co-infection. Using two host species we investigate how an increasing pathogen dose and co-infection (with two bacterial strains differing in their virulence), affect the virulence of this host-generalist pathogen in two phylogenetically distant host species. Our aim is to shed light on how the infective doses and co-infections in opportunistic pathogens shape the disease outcome in different host species, and thus increase the present understanding of disease evolution and how disease epidemics emerge in differing conditions. As an infection model we use the opportunistic fish pathogen *F*. *columnare*, and as hosts the rainbow trout (*Oncorhynchus mykiss*), a Salmonid host frequently infected in fish farms, and the zebra fish (*Danio rerio*), a Cyprinid host.

## Materials and Methods

### Pathogen

*Flavobacterium columnare* is a globally important fish pathogen in freshwater aquaculture [19, 20] and known to affect several fish species in fish farming and in the wild as a causative agent of columnaris disease [21, 22, 23]. The common clinical signs of the disease include gill necrosis, fin erosion and skin lesions such as the typical saddleback symptom around the dorsal fin [20, 24]. The disease is transmitted from infected fish via water and biofilms [25, 26]. In Europe, *F*. *columnare* is an especially difficult pathogen in salmonid fish farming, where it can cause severe fish mortality within the rearing units [20, 25]. The disease outbreaks occur in the summer when the water temperature naturally rises above 20°C [27]. Two previously isolated *F*. *columnare* strains were used in this study: a high-virulence strain B185 isolated during a columnaris disease outbreak at a salmonid fish farm in Central Finland (farm L, see details on the strain isolation and virulence in our previous studies [28, 29]) and a low-virulence strain B398 isolated from the inlet water of another salmonid fish farm in the same area (farm V, see [25]). Pure cultures were stored frozen at -80°C in a stock containing 10% glycerol and 10% fetal calf serum. For the experiments, the bacterial strains were grown in modified Shieh medium [30] at 26°C with constant agitation (150 rpm).

### Host species

The rainbow trout is a cold-adapted fish species, occurring naturally in the Pacific Ocean and cold streams in the North American continent from Alaska to Mexico [31]. After introduction into Finland around 1900, the rainbow trout has become the most important commercially farmed fish species in the country [32], and since the 1990’s has been severely affected by columnaris disease during warm water periods [20]. As *F*. *columnare* is prevalent at salmonid farms and their inlet waters in Finland, we used rainbow trout as a model species representing a natural host of *F*. *columnare*. For the study, apparently healthy fingerling rainbow trout with no known history with *F*. *columnare* were obtained from a stock of a fish farm (farm V) in Central Finland. The fish were obtained from the farm during a cold water season (when no outbreaks occur), brought to our fish rearing facilities where *F*. *columnare*-free well water is used, and maintained for two months at 15.0–16.0°C before conducting the experiments. The average weight of the fish was 1.25 g.

The zebra fish is a well-established laboratory animal that shares the temperature optimum of the pathogen (for zebra fish, see [33]; for *F*. *columnare*, see [24]). It is a tropical species indigenous to South Asia, and thus it does not have a recent co-evolutionary history with the bacterial strains used in this study. The adult, unsexed, disease-free zebra fish (average weight 0.21 g) were obtained from Core Facilities (COFA) and Research Services of Tampere (University of Tampere, Finland).

Both rainbow trout and zebra fish have been previously used as experimental hosts for *F*. *columnare* [21, 25, 34, 35], but how the bacterial strain and the dose affect the onset of columnaris disease has not yet been thoroughly studied. If the zebra fish are found to respond to the experimental columnaris infection in a similar way to the rainbow trout, they could be used as a reliable model in further columnaris disease experiments.

### Infection treatments

To examine the interactions between virulence, infection dose, and host species, the fish were infected with the two *F*. *columnare* strains, and with a 1:1 mixture of these strains, by bath challenge [29]. The fish were individually challenged in 50 ml of aerated ground water with 5.×10^5^, 1.0×10^6^, 3.0×10^6^, 6.0×10^6^, 9.0×10^6^, 1.2×10^7^, 1.6×10^7^, 2.0×10^7^ and 3.0×10^7^ CFU (colony forming units) ml^-1^ of overnight-grown bacteria for 2 hours at 25°C in two fish per dose. The dose was treated as a continuous variable, totaling 18 replicate fish per species per each treatment group (high-virulence strain, low-virulence strain and co-infection). Per species, 5 replicates of negative control fish (sham-exposed to sterile Shieh medium) were used. After being challenged, the fish were transferred individually into 1 liter aquaria with 0.5 liter of ground water, and monitored for clinical signs of disease and morbidity for 5 days, the first 48 hours at 2-hour intervals. The water temperature was maintained at 25.0–26.3°C throughout the experiment. The fatally moribund fish were euthanized by decapitation. Also the surviving and the control fish were euthanized in the end of the experiment. To verify the columnaris infection, cultivations from gills were spread on Shieh agar supplemented with tobramycin [36]. The yellow colonies with the rhizoid morphology typical to *F*. *columnare* were considered as an indicator of columnaris infection. The experiment was conducted under permission ESAVI-2010-05569/Ym-23, granted by the National Animal Experiment Board at the Regional State Administrative Agency for Southern Finland.

### Statistical analysis

The data were analyzed using a generalized linear model (GLM) for binomial distribution. Two factors (‘host species’ and ‘treatment’ (high-virulence strain, low-virulence strain or co-infection)), a continuous covariate (‘dose’), and all their possible interactions were included as variables to explain the fate of the fish (dead or surviving) within the time from the beginning of the experiment (see [37]). All the infected rainbow trout ‘died’ during the experiment. The last moribund rainbow trout were euthanized 42 hours before the end of the experiment. As the gill cultures taken from the infected and moribund fish were positive for *F*. *columnare* and cultures from the surviving fish were negative, it can rather safely be assumed that zebra fish surviving up to 141 hours (i.e. until the end-point of the experiment) were able to resist, or tolerate and survive, the infection. Thus, we did not consider the surviving individuals as censored cases. The model selection was based on Akaike information criteria (AIC; Table 1) and the analysis was conducted with the software R 2.15.2 and the package Lme4. When interpreting the effects of the terms included in the model, a significance level of 0.05 or less was used.

**Table 1:** Model selection based on Akaike information criteria (AIC).

Model	AIC	df	P
host*treatment*dose	234.23		
**host+treatment+dose+host:treatment+host:dose+dose:treatment**	231.22	2	0.611
**host+treatment+dose+host:treatment+dose:treatment**	230.72	2	0.173
**host+treatment+dose+host:treatment**	232.93	2	0.104
**host+treatment+dose+dose:treatment**	236.78	1	0.009

The model with smallest AIC value estimating the morbidity risk of the host (rainbow trout or zebra fish) within time is underlined.

The degrees of freedom (df) and significance levels (P) are given for the goodness of fit compared to the next higher level model.

Single- and co-infections are included in the term ‘treatment’.

## Results

**Fig 1:**
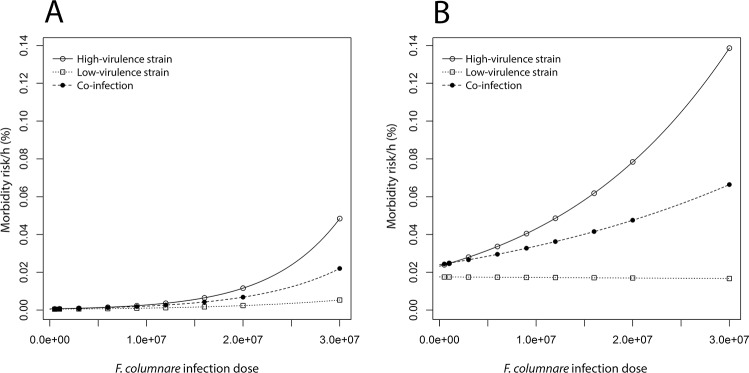
Estimated mortality risk per hour of A) zebra fish (*Danio rerio*), and B) rainbow trout (*Oncorhynchus mykiss*) infected with a high-virulence (continuous line) and a low-virulence (dotted line) strain of *F*. *columnare*, and their mixture, i.e. co-infection (dashed line).

The risk of fatal infection of the host was significantly influenced by the dose, the treatment (high-virulence strain, low-virulence strain or co-infection) and the host species (Fig 1, Tables 2 and 3). We found that 1) the increase in the dose correlates positively with the host morbidity risk when the hosts are exposed to the high-virulence strain or the mixture; 2) the infection success in the co-infected hosts is approximately an average of that of hosts infected with the high-virulence and the low-virulence strains (Fig 1), indicating that only the high-virulence strain is responsible for the host morbidity; and 3) the rainbow trout is more susceptible to the columnaris infection than the zebra fish (Fig 1), but both hosts respond to the bacterial doses and strains qualitatively similarly. All the moribund hosts were found positive for *F*. *columnare* in bacterial culture taken from fish gills, whereas the unexposed hosts and hosts surviving the infection were found negative.

**Table 2:** The significance and test values of the bacterial dose, the treatment and the host species on the morbidity risk of the hosts.

Source	Df	Deviance	Residual deviance	P
**Host**	1,106	84.350	116.663	**<0.001**
**Dose**	1,105	9.606	107.057	**0.002**
**Treatment**	2,103	14.654	92.403	**<0.001**
**Host:Dose**	1,102	10.205	82.198	**0.002**
**Dose:Treatment**	2,100	4.522	77.677	0.104

Significant P values are denoted in bold.

**Table 3:** The effect of the bacterial dose, the treatment and the host species on the host morbidity risk.

Source	Estimate	SE
**(Intercept)** ^**a**^	-3.738	0.390
**Host(Zebra fish)**	-3.633	0.571
**Dose**	6.372^−8^	2.805^−8^
**Treatment(Co-infection)**	3.337^−2^	0.505
**Treatment(Single infection, low)**	-2.861^−1^	0.496
**Host(Zebra fish):Dose**	8.272^−8^	3.197^−8^
**Dose:Treatment(Co-infection)**	-2.832^−8^	3.353^−8^
**Dose:Treatment(Single infection, low)**	-6.542^−8^	3.186^−8^

^a^ Intercept includes the effects of the host (rainbow trout) and the treatment (single infection, high).

## Discussion

Opportunistic pathogens are often host-generalists and may survive and replicate outside the host [3], thus having different environmental dynamics than obligate pathogens. Pathogens that are durable in the outside-host environment may not have high fitness costs related to virulence [38], which has also been observed for *F*. *columnare* [39, 40]. The ability to survive and replicate outside the host can contribute significantly to the infective bacterial populations in the environment, and therefore information on the relationship between the number of free-living bacteria (the infection dose) and disease virulence is needed. Although the influence of dose on disease dynamics has been widely acknowledged, its effect on parasite virulence and reproduction is sometimes unclear or even contradictory [14]. Additionally, the experimental evidence from the infective doses of opportunistic pathogens is scarce, especially in different host species. We addressed these issues by infecting two host species with increasing doses of a high-virulence and a low-virulence bacterial strain in a single and in a co-infection.

We found a strong positive relationship between the dose and the host morbidity risk in the treatments in which the high-virulence strain was involved (Fig 1). As the host morbidity risk in this study is a measure of pathogen virulence (as demonstrated in e.g. [2, 41, 42, 43]), our result suggests that the virulence of *F*. *columnare* is strongly dose-dependent. This finding is in agreement with the experimental evidence from obligatory pathogens [14, 44]. Interestingly, in contradiction with numerous previous studies [5, 7, 45, 46, 47], we did not observe any additive effects of co-infection on the host morbidity risk. This indicates that the outcome of the infection in this study is affected by the interplay between the bacterial strain and the dose. Previous studies (e.g. [48, 49]) have shown that the more virulent strains have a competitive advantage in mixed infections, whereas in some systems, like *Schistosoma mansoni*, co-infections may favor the less virulent strains [50]. Our result suggests that the presence of a low-virulence strain may significantly alter the co-infection outcome, most likely by diluting the infection dose. Indeed, if the low-virulence strain lacks the ability to produce essential virulence factors needed for a successful infection, its presence may reduce the total severity of the disease outbreak. This is an important finding as the interactions between high-virulence and low-virulence strains are generally poorly understood. Yet, pathogen strains with variable levels of virulence often co-occur in the environment [20, 22], thus influencing the onset of disease outbreaks or host immune response.

Also interference competition via antimicrobial compounds like colicins (i.e. inhibitory compounds targeted to hamper the growth of other conspecific strains) may have trade-offs with virulence [51]. *F*. *columnare* has been reported to produce bacteriocins that are equivalent to colicins, as demonstrated in [52]. However, in order to find out if the mechanism leading to reduced virulence in our system builds upon the competitive interactions between the bacterial strains, more studies are needed in the context of virulence evolution.

Although maintaining the ability to infect multiple host species can be an efficient survival strategy, it may result in a trade-off, leading to lower pathogen virulence [11, 12, 53]. We found the two host species to respond qualitatively similarly to the increase in the infection dose, but the rainbow trout was more sensitive to the increase than the zebra fish. Similar associations between *F*. *columnare* strains and the host species have also been found in salmonids in general and in channel catfish [35, 54, 55]. However, more extensive studies on a variety of host species would be needed to find out if our results are due to adaptation of the strains to the rainbow trout, or if host generalism has trade-offs with virulence of *F*. *columnare*. Nevertheless, our result has important implications because *F*. *columnare* populations encounter a wide range of host species both in the wild and at fish farms [21, 22, 23, 56].

The sensitivity of the rainbow trout in this study can also be partly caused by the experimental conditions. The water temperature during the experiment was not optimal for the cold-adapted rainbow trout, although it still was within the temperature range naturally occurring in fish farming conditions. Indeed, columnaris disease outbreaks at fish farms are typically prevalent during the warm water season [20, 57]. Yet, our findings confirm that zebra fish is a suitable model species for experimental studies of *F*. *columnare* infections. Zebra fish has been successfully used as an infection model for columnaris disease already in prior studies [34, 35], but the infection dynamics of *F*. *columnare* in zebra fish compared to rainbow trout has remained unclear. Information about the comparability of the dose responses in these two species is therefore intensely needed to be able to replace the stress-sensitive rainbow trout in the demanding laboratory experiments. Unlike the rainbow trout, the zebra fish is well suited to laboratory conditions; it is a small-sized species that thrives in warm temperatures (as does the pathogen) and does not require constant water flow [33]. Additionally, zebra fish are available year-round.

Our results suggest that an increase in dose can lead to more severe disease and poorer host survival in host-generalist opportunistic pathogens, but the host survival may be dependent on the original ability of each bacterial strain to cause disease in a strain-specific manner. For the same reason, different pathogen strains may not necessarily have additive effects on disease virulence. Based on our results, it seems that the interactions between the dose and the pathogen strains are important drivers of infection in different host species, and warrant for more studies for evolution of virulence and pathogen host range. Furthermore, from an applied perspective, using zebra fish as an infection model can provide valuable information on the virulence of *F*. *columnare*, as the zebra fish shares the temperature optimum of the pathogen and tolerates the experimental conditions well.
